# Planting a seed - child health care nurses’ perceptions of speaking to parents about overweight and obesity: a qualitative study within the STOP project

**DOI:** 10.1186/s12889-019-7852-4

**Published:** 2019-11-09

**Authors:** My Sjunnestrand, Karin Nordin, Karin Eli, Paulina Nowicka, Anna Ek

**Affiliations:** 1 Division of Pediatrics, Department of Clinical Science, Intervention and Technology, Karolinska Institutet, Stockholm, Sweden; 2 Unit for Biocultural Variation and Obesity, Institute of Social and Cultural Anthropology, University of Oxford, Oxford, UK; 3 Division of Health Sciences, Warwick Medical School, University of Warwick, Coventry, UK; 4 Department of Food Studies, Nutrition, and Dietetics, Uppsala University, Uppsala, Sweden

**Keywords:** Children, Overweight, Obesity, STOP project, Nursing, Primary care, Qualitative research, Thematic analysis

## Abstract

**Background:**

Nurses in child health care (CHC) centers in Sweden play a key role in the early detection and management of childhood overweight/obesity, through meeting families early, regularly and over many years. However, research focusing on CHC nurses’ perceptions of their role in childhood overweight/obesity management is scarce. As part of the EU-funded project “Science and Technology in childhood Obesity Policy” (STOP), this study examines CHC nurses’ perceptions of speaking to parents about children’s overweight/obesity and of their role in referring children to treatment for overweight/obesity.

**Methods:**

All registered CHC nurses in Stockholm County (*n* = 442) received an email invitation letter explaining the study. Individual face-to-face visits (*n* = 15) in selected centers, and phone calls (*n* = 24) to nurses working in these centres were also conducted. In total, 17 CHC nurses (all female, average work experience 6.7 years (SD ± 4.9 years)) from 10 municipalities (including four of the top five municipalities with the highest prevalence of overweight and obesity) in Stockholm County were interviewed. The interviews were conducted by phone, transcribed and analyzed using thematic analysis.

**Results:**

Two main themes were developed through the analysis: 1) *The relationship between the nurse and the parent* and 2) *Glitch in the system.* Under the first theme, nurses reported that weight-related discussions were facilitated by building and maintaining trust with parents. However, nurses also said they were reluctant to address children’s weights if this could compromise parents’ trust. Under the second theme, nurses highlighted several organizational barriers to addressing a child’s weight with parents, including insufficient cooperation with other healthcare providers and limited time for visits. Nurses also identified lack of sufficient knowledge about what to offer the family and lack of confidence in their communication skills as additional barriers.

**Conclusions:**

We found that pediatric nurses perceive relational and organizational factors as barriers to address childhood obesity with parents. To improve care, it is necessary to provide nurses with continuing education about obesity and communication skills and organizational support to improve communication within the healthcare system.

**Trial registration:**

ClinicalTrials.gov NCT03800823; 11 Jan 2019, prospectively registered.

## Background

In a healthcare setting, bringing up child overweight in conversation with parents can be difficult. Parents often do not recognize that their children have overweight or obesity, which hinders the initiation of weight management [1, 2]. Even when parents acknowledge a child’s high weight status, they may be reluctant to engage in weight management, because many parents reframe their children’s overweight as a ‘chubbiness’ they will outgrow [3]. However, in the clinical encounter, healthcare providers may be as reluctant as parents to acknowledge verbally a child’s overweight or obesity. According to previous studies, healthcare providers feel uncomfortable when initiating discussions about child overweight because of worries about offending parents [4] and awareness of the prevailing societal stigma attached to excess bodyweight [5]. However, healthcare providers’ reluctance to discuss overweight and obesity may lead children to miss opportunities for treatment; as one study found, healthcare professionals and parents often prefer to wait until the other party raises the issue, thereby leading to stalemate [6]. Child overweight tends to be an even more sensitive topic if the parents themselves have overweight or obesity [4]. Because communication between parents and healthcare professionals is key to the management of children’s overweight/obesity, it is important to understand why healthcare professionals may be reluctant to speak about overweight and obesity among young children.

In Sweden, CHC is free of charge, and 98–99% of all families attend regular visits from birth to 5 years of age [7, 8]. Families are seen by CHC nurses frequently throughout the child’s first year: approximately every other week during the child’s first 2 months, and then every other month. When the child turns 1 year old, CHC visits are scheduled annually [9]. During each visit, the child’s growth is monitored using weight and height growth charts, and families receive customized support and monitoring of the child’s physical and psychological development [10]. Thus, CHC nurses are the main point of contact for the healthcare of young children, routinely providing families with at least 15 visits; in comparison, families of young children attend only three to five physician visits [11].

CHC nurses thus play a pivotal role in the early detection of rapid weight gain. According to the standard care protocol, when overweight is identified, families should receive support from the CHC nurse; the nurse can also refer the child to a dietician or physiotherapist if the family needs more support. Referrals to outpatient pediatric clinics are recommended only to children who have obesity and are at least 4 years of age [12]. Despite this well-developed structure, when we recruited families to an obesity treatment study through CHC centers (*n* = 68) in Stockholm County, we found it challenging to identify enough participants [13]. In fact, data from the Swedish National Outpatient Register confirm that very few children under the age of 4 are diagnosed with obesity and referred to treatment [8].

According to a recent meta-synthesis, when attempting to discuss child weight with parents, the barriers and facilitators healthcare professionals encounter have been mostly intra- or interpersonal [14]. Interestingly, on an organizational and societal level, only barriers have been identified [14]. Further, the findings by Bradbury et al. are largely in line with an earlier synthesis of the field from 2007 [15] showing that healthcare providers should expect a wide range of reactions when communicating with parents about children’s weights. Many parents avoid talking about child overweight because they worry it may lead to an unhealthy weight fixation [16] and affect the child’s self-esteem and emotional well-being [14, 15, 17, 18]. In other cases, however, concerns about children’s emotional wellbeing may lead parents to seek help from healthcare providers [4]. The CHC nurses, on the other hand, may be reluctant to communicate with parents about child weight because they feel they lack skills in child weight management [14, 19], or because a CHC visit includes other priorities (such as vaccination or vision screening) which limits a comprehensive assessment and discussion of weight-related topics [19], or because they feel frustrated about inconsistent guidelines regarding referral to treatment [14, 20]. Moreover, even when CHC nurses refer children to obesity treatment, physicians sometimes dismiss their concerns about the child’s weight [11, 21–24].

With regard to factors that facilitate communication about child overweight with parents, nurses identified feeling confident in their professional skills [14, 20], experiencing positive parental responses to mentions of child overweight, and engaging in conversation after parents initiate the topic [14, 21] or welcome the support offered by the nurse [11, 14] as particularly helpful. Importantly, when parents initiate conversations about the child’s overweight, nurses worry less about being perceived as critical and judgmental [6]. Because nurses consider a trusting relationship with parents as foundational to their work, they highly value being able to discuss children’s overweight in a non-judgmental way [14].

Understanding how CHC nurses perceive communication about child overweight/obesity is crucial to designing and testing childhood obesity interventions in Sweden. As part of the EU funded project “Science and Technology in childhood Obesity Policy” (STOP), we are now testing whether a family-based childhood obesity treatment [13] is feasible when offered to families with children from 2 years of age and to children with both overweight and obesity [25]. Because families will be recruited from CHC centers, it is essential to understand barriers and facilitators that may influence how nurses address child overweight and obesity at such young ages. Previous evidence on that matter is becoming dated; the two studies on CHC nurses’ experiences with weight-related communication [4, 11]; were published in 2013 (with data collected in 2010–2012). The CHC system has experienced a number of changes since these earlier studies were published, and CHC nurses now have a more substantial role in the early detection and management of child overweight/obesity, such that an update is needed to inform our planned treatment feasibility study as well as the literature more broadly. Thus, this study aims to explore CHC nurses’ perceptions of speaking to parents about children’s overweight/obesity and of their role in referring children to treatment for overweight/obesity.

## Methods

### Participant recruitment

Participants were recruited through a purposive sampling approach. First, an invitation letter explaining the aim and content of the study was sent by email to all registered CHC nurses employed in Stockholm County (*n* = 442). Nurses who wanted to participate in the study were asked to contact the research group. No nurses responded to this letter. As the next step, the first author, MS, visited 15 CHC centers, most of which were located in areas with a high prevalence of childhood overweight and obesity, and provided information about the study in person to all nurses at each CHC center. All nurses who attended those meetings were invited to participate in an interview. In addition, nurses (*n* = 24) from 8 of the visited 15 CHC centers were individually approached by phone. The nurses who declined participation reported they had limited experience of addressing children’s overweight to parents. Nurses who wanted to participate were sent an informed consent form by mail and were asked to send the signed original back to the research group. The interviews were then scheduled for a date and time that suited the nurses.

### Semi-structured interviews

Data were collected using individual, semi-structured interviews. All interviews were conducted by the first author, MS, as part of a Master’s degree. Interviews took place over the telephone, except one that was held in the nurse’s office due to the nurse’s preference for an in-person interview. The interviews were audio recorded and transcribed verbatim by MS and a trained journalist. Field notes were made during the interviews.

The interviews followed an interview guide developed by MS in consultation with an expert group (all female). The expert group consisted of main supervisor AE, a postdoctoral fellow in early childhood obesity treatment, co-supervisor PN, a professor in Communication of Dietetics, and co-supervisor KN, a pediatric nurse and research assistant. All three experts have extensive experience of talking to families about overweight and obesity and have worked closely with child health professionals in different settings to improve overweight and obesity care. The interview questions aimed to capture CHC nurses’ experiences of communicating with parents about their children’s overweight, and to identify the nurses’ perceived barriers and facilitators to referring children to obesity treatment (see Table 1). The interviewer asked all questions to all nurses, as well as individualized follow-up questions based on each nurse’s responses. The questions were pilot tested using cognitive interviews [26] with two CHC nurses and then revised for a final version. During these cognitive interviews, think-aloud and verbal probing techniques were used. Think-aloud is a technique where the participant is encouraged to verbalize how she/he reasons when answering the questions and verbal probing refers to follow-up questions asked by the interviewer. Both techniques enable the researcher to identify strategies used by the participant when she/he attempts to answer the question to gain a better understanding of the cognitive processes evoked by the questions [26]. It was emphasized that the participants were free to express their own thoughts and to raise additional issues during the interviews. Questions that did not provide comprehensive answers or did not capture what was intended were removed or rephrased. For example, the question “*When do you usually address children’s overweight?”* was removed because the answer was given in a previous question. In another example, the question “*Do you feel that the children’s weight status is perceived as a problem for parents?”* was changed to “*When do parents seek help for their child’s obesity?”* In addition, some words were changed or added to questions in order to soften the tone. The final version of the interview guide consisted of 14 open-ended questions, see Table 1.

**Table 1:** Questions in the interview guide asked to all nurses

1	How do you usually tell parents that their child has overweight?
2	Many people feel that children’s overweight is a sensitive subject to talk about. How do you feel about talking about children’s overweight?
3	Think of a conversation where you expressed concern over a child’s overweight that you felt was successful. Can you tell me about that conversation?
4	Think of a conversation where you expressed concern over a child’s overweight that you felt was less successful. Can you tell me about that conversation?
5	If children are present during these conversations, in your experience, how does it affect the conversation?
6	We know that it can be difficult for parents to see their child as having overweight or obesity. How do you perceive parents’ awareness regarding their children’s weight status?
7	When do parents usually seek help for their child’s overweight?
8	If during a meeting with a parent you find out that the parent has a difficult life situation, how do you proceed to support the parent?
9	How is a conversation about a child’s weight affected by who is present at these visits?
10	What kind of support and advice do you offer to parents?
11	What do you think enables parents to accept your support and advice?
12	To what extent do you feel that you have the opportunity to affect lifestyle changes?
13	Why do you think it is difficult to make families engage in studies about childhood obesity?
14	I do not have any more questions, but is there anything you would like to add?

The reporting of this study follows the COnsolidated criteria for REporting Qualitative research (COREQ) checklist, see Additional file 1.

### Thematic analysis

The interviews were analyzed using thematic analysis, following a realist approach. This approach allowed us to focus on the experiences the participants described, ascertain the meanings they assigned to these experiences, and relate these to the everyday realities of working in CHCs [27]. The transcribed interviews were read and re-read by MS and KN and coded by MS using an inductive approach, without being limited to a pre-existing coding frame [27]. Thus, identified themes were associated with the participants’ responses rather than directly linked to the specific questions asked. MS noted initial ideas that emerged while reading through the interviews, to identify an overall pattern. MS then placed relevant textual entities into a new document and coded these. MS, AE and KN met weekly to follow the progress of analysis and discuss the coding. Once the coding was completed, MS, AE and KN sorted the codes into different themes and subthemes. Themes were identified on semantic level (i.e., each theme reflected content explicit in the data) [27]. Lastly, all identified themes were organized in a table.

## Results

A total of 17 CHC nurses representing 10 CHC (all female with an average working experience of 6.7 years (SD ± 4.9 years)) were interviewed. The interviews lasted on average 48 min (SD ±18 min, min 22 min – max 93 min. In Stockholm County, the overall mean prevalence of overweight is 8.8% and obesity is 1.8%, based on measurements of 25,581 4-year-olds, representing 89.8% of all children in the county [28]. Two out of the 10 CHC centers represented areas with lower prevalence (the lowest prevalence in centers included in this study was 7.4% for overweight and 1.2% for obesity). Four CHC centers represented areas in the top five for prevalence of childhood overweight and obesity in Stockholm county (with the highest prevalence of overweight being 11.8% and obesity being 4.6%). Most of the areas with the highest prevalence of overweight and obesity were also the most socioeconomically disadvantaged, with higher unemployment rates and a larger immigrant population. Specifically, the lower purchasing power in these areas was related to the prevalence of obesity according the recent report from the CHC [28]. The number of interviews conducted in our study (*n* = 17) agrees with previous research which suggests that nine interviews are sufficient for data saturation, and that 16 interviews are needed to gain a comprehensive understanding of issues raised in interviews [29].

### Themes from the thematic analysis

**Fig. 1:**
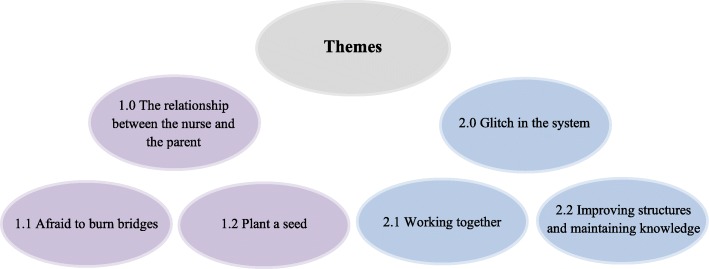
Schematization of identified themes from the thematic analysis

Two main themes and four subthemes were developed through the thematic analysis. The main themes were 1) *The relationship between the nurse and the parent*, and 2) *Glitch in the system*. The themes and subthemes are illustrated in Fig. 1 and described in detail below.

#### The relationship between the nurse and the parent

Nurses emphasized the importance of developing and maintaining trusting relationships with parents (subtheme 1.1; afraid to burn bridges), and interacted carefully with parents to raise awareness of child overweight (subtheme 1.2; plant a seed).

##### Afraid to burn bridges

All nurses reported that overweight was a sensitive topic. Although reactions to weight-related conversations differed between parents, with some parents responding receptively and expressing motivation to change their lifestyle behaviors, many nurses felt uncomfortable raising issues regarding a child’s weight with parents. They acknowledged that this was partly due to fear of offending parents: *“They come here with, with their little... little children who they adore, and think are perfect, so it’s all about... to avoid offending them.” (R1)* Indeed, many nurses experienced some parents reacting strongly to the information that their child has gained too much weight. Parents could get upset, angry or showing that they felt accused. Some nurses felt attacked by parents; others felt misunderstood because they felt it was not their intention to upset the parents, and some said they even began to question their own skills and reflected on whether they could have done something differently.

Central to the nurses’ concerns about upsetting parents was the need to maintain a trusting relationship. Nurses said that trust is gradually established over time, and that a trusting relationship with parents is carefully built over multiple CHC visits. A trusting relationship was so important to some nurses that they preferred to avoid initiating a conversation about a child’s overweight rather than risk losing the parents’ trust, as one nurse explained: *“There, I don’t bring this up [the child’s overweight] … because this is so sensitive for the mother.... I don’t want to, and it has to do with trust in a way, you see, I want this mother to feel calm and secure with me, so I feel that I almost sabotage that relationship if I would go on with overweight there [talking about the child’s overweight].” (R5)* According to some nurses, if the parent had overweight, the conversation was perceived as even more difficult. As this nurse also described: *“I can completely relate to myself, because I know how it feels to be constantly nagged on because you are overweight … and have other’s eyes on you all the time…”(R3)* Nurses also avoided mentioning a child’s overweight if the family faced difficulties or seemed stressed during the visit. In these cases, the nurses deferred the conversation for a follow-up visit a few months later, in hopes that the family would be better positioned to have the conversation then. The nurses explained that avoiding or deferring conversations allowed them to maintain rapport: if their relationships with parents were damaged, parents would decline future visits at the CHC center.

##### Plant a seed

Many nurses said that parents did not always recognize their child had overweight. In fact, they said that many parents considered higher weight in young children as a sign of health, believing their child will eventually outgrow it. Parents’ perceptions were therefore barriers to addressing weight issues at the CHC centers: *“It’s somehow that we are at the same level; that they [the parents] understand what the problem is. Because if they don’t understand it, they will not do anything about it either. I wouldn’t do so either - if I didn’t consider something to be a problem. Because that’s how we work… we don’t fix things we think is alright.” (R5)*

Some nurses stated that they would bring up the child’s overweight regardless of the parents’ readiness to discuss it, because they felt it was their responsibility. As one nurse described it, “*there are many situations … that can arise where one needs to be uncomfortable or bring up something that perhaps the parents don’t agree with. My role is to stand up for the child if I see a difficulty or so.” (R9)* However, most nurses emphasized the importance of communicating carefully with parents. In some cases, careful communication was developed through multiple visits and the building of trust. One nurse said that when she got to know the parents, she knew which approach would “usually work” for them. Another nurse said that previous positive experiences of raising sensitive topics with the family improved communication. Other nurses expressed that if parents trusted their nurse, they were more likely to take the nurse’s concern about the child’s weight status seriously.

To achieve careful communication, a common strategy the nurses used was to open the conversation by asking the parents open-ended questions. For instance, the nurses used the child’s BMI growth charts to show the parents the child’s weight development and then asked “*what are your thoughts regarding your child’s weight development since the last time we met?*”. Many nurses agreed that parental reactions determined how they chose to proceed with the conversation and how they tailored their approach towards the parents. Some nurses described it as an interplay with the parents where they either approached them straightforwardly or backed off if they noticed that the parents were offended. However, regardless of the outcome of the conversation, the nurses said that they at least could initiate a thought, “*plant a seed*” *(R14),* which would enable a conversation about the child’s weight during the next visit. *“Those who are the most upset are perhaps the ones you should talk to the most. But it might be there where you back off, because you realize that here, you’ve reached a dead end. And there, it’s back to this trust again. You want to get the child back, you want to continue to follow the child. Approach them in the wrong way and they - yes, some change to another CHC center.” (R6).*

#### Glitch in the system

Nurses identified several organizational factors that interfered with their ability to communicate with parents about children’s weights and offer them appropriate support. The subthemes focus on organizational and relational factors in the healthcare system (subtheme 2.1: working together, subtheme 2.2: improving structures and maintaining knowledge).

##### Working together

Nurses felt that good cooperation with other healthcare providers enabled them to stress the importance of children’s weight management to parents, saying that if the same message came from more than one professional source it was more likely that the parents would accept the support offered. However, most nurses said better cooperation with other healthcare providers, such as dieticians, was still needed for onward referrals. A few nurses pointed out that childhood overweight was not their area of expertise and suggested that families should be provided with better access to dieticians. One nurse further suggested that CHC centers should collaborate with preschools, considering the amount of time the children spend there in comparison to their limited CHC visits: *“I wish we had much better cooperation with preschools about how they eat there ... because I think, we meet the child like... yes, if everything is pretty good then we meet them for half an hour each year, when they are in these slightly older ages. While the preschools meet them every day.” (R5).*

Inconsistent cooperation within the healthcare system discouraged nurses from raising the topic of child weight with parents. For example, without clear guidelines on how to treat children with overweight, one nurse said she felt unsure about when to offer support to children with overweight, where to refer children, and whether she had the ultimate responsibility, and was therefore reluctant to begin a conversation on this. Nurses also said communication between CHC centers and pediatric clinics was lacking, and that clinics sometimes rejected their referrals for obesity treatment because the nurses’ concern about children’s overweight was not shared by the pediatricians. As one nurse expressed: *“Then it gets the other way around, then it’s me who have... raised this as a problem, although it may not be one … or the doctor said it wasn’t. I had one such case – I thought it was really sad, then you get a little tired, then you feel… why should you work with this when you don’t get any support.” (R8)* Additionally, some nurses were unsure about how families would be treated at the pediatric clinic following referrals for obesity treatment. One nurse revealed she felt reluctant to refer children to the pediatric clinic because she met parents who had been treated poorly at the pediatric clinic. She was worried, she said, that a referral that resulted in poor treatment would undermine her efforts to convince parents to accept support: *“That’s really difficult. You’ve been talking, or maybe had several follow-up visits and really struggled to get the parents on the right track - and then you finally send the referral and then they are poorly treated at the pediatric clinic. It makes you feel quite abandoned.” (R8)* Facing such organizational barriers, nurses often felt their efforts to speak to parents about children’s overweight/obesity would be useless.

##### Improving structures and maintaining knowledge

Because CHC visits needed to cover several areas of child health, time constraints sometimes led nurses to defer or cut short a conversation about a child’s overweight. For example, one nurse said that time constraints meant she tended to rush through a conversation rather than to listen to the family’s needs. Most nurses wished they could spend more time with each family, with longer visits and more follow-up appointments. As one nurse explained: *You have a conversation [about the child’s overweight] then you see the family a year later and then it [the child’s overweight] looks better, or it looks even worse.” (R11)* Nurses perceived lack of time as particularly challenging if parents were unaware of the child’s overweight or if parents did not agree that the child’s weight status was something to worry about.

Because of the sensitivity of discussing children’s overweight and obesity, some nurses felt it would be valuable to inform the parents in advance that the child’s growth will be discussed during the visit. The nurses reasoned that if the parents were prepared, they were less likely to become upset. In addition, a few nurses noticed that parents do not reflect on the child’s normal growth development. Parents seem unaware that children are not expected to gain weight at the same rate after 1 year of age, as one nurse explained: “*We reward and praise [the parents] when they [the children] gain weight and then all of a sudden we say the child has gained too much weight and it’s difficult for the parents to keep up. We need to prepare the parents in a better way to make this conversation easier.” (R2)* Another nurse further suggested that all families, regardless of the child’s weight status, should be offered a routine visit to discuss diet and lifestyle habits. She believed that this could facilitate a discussion about children’s weight without parents feeling judged or blamed. Knowledge and confidence in communication skills also made it easier for nurses to speak about children’s overweight with parents. While nurses obtained some knowledge about childhood overweight and obesity during their nursing education and CHC training, they said their practice would benefit from regular updates on new research in the area; for example, knowing more about the importance of children’s weight management at young ages would allow nurses to use research evidence when speaking with parents. One nurse also called for more knowledge about the genetic factors influencing the risk of developing overweight and obesity, as it would allow nurses to convey to parents that they are not to blame and are not being judged: *“We have very different genes for this [overweight]… I don’t know much about that but it’s obvious… It’s much more difficult for some families than others.” (R8)*

## Discussion

In this study, we interviewed CHC nurses in Sweden to explore their perceptions of communicating with parents about young children’s overweight. We chose to focus on CHC nurses in recognition of their central role in the early identification and prevention of overweight and obesity among young children. Through the analysis, we developed two overarching themes. Under the first theme, *The relationship between the nurse and the parent*, nurses reported that weight-related discussions were facilitated by building and maintaining trust with parents. However, nurses also said they were reluctant to address children’s weights if this could compromise parents’ trust. Under the second theme, *Glitch in the system,* nurses highlighted several organizational barriers to addressing a child’s overweight and obesity with parents, including insufficient cooperation with other healthcare providers, limited time during the visits, limited knowledge about childhood obesity and lack of sufficient communication skills.

Our findings confirm that CHC nurses highly value the development and maintenance of trusting relationships with parents, and that this may lead nurses to avoid bringing up issues related to children’s weights [11]. However, pervious research has found that parents prefer to receive information about child weight status rather than avoid discussing it [21, 30]. Healthcare providers also believe that parents *expect* to be informed about their child’s weight development [24, 31]. Indeed, a previous study in Sweden has shown that, in most cases, CHC nurses, rather than parents, were those who initiated weight status conversations [11]. Our study, however, found that CHC nurses would prefer if parents initiated conversations about children’s weights. The findings also suggest that CHC nurses’ confidence in communication about children’s weights has not increased since the first studies on the topic were published in the early 2010s [4, 11, 19].

Earlier research has found that strong relationships between parents and clinicians are crucial to long-term weight management for children with obesity [11, 32]. Dev et al. called it “partnerships in which parents and providers join together for promoting child health” [32]. Paradoxically, however, nurses felt that raising issues related to children’s weight status risked undermining this relationship, ultimately affecting their ability to support the family appropriately [21, 32]. This risk was fueled by parents’ lack of awareness of their children’s weight status [22]. Previous studies conducted in Europe and North America, involving parents of diverse ethnic and socioeconomic backgrounds, have found that parents tend to underestimate their children’s weight status. This has been identified as one reason why parents do not initiate weight management [23]. This lack of awareness, moreover, is compounded by weight stigma: studies conducted in the US, the UK and Sweden have found that parents of children with overweight or obesity often feel guilty and fear being judged by others [20] due to the social stigmatization of overweight and obesity, which is well documented [3, 24, 25]. A common prejudice in clinical and public health practice is the belief that stigma will motivate individuals to lose weight; however, research has proven that stigma exacerbates chronic stress, emotional eating, and obesity [3, 24, 25]. Our findings show that weight stigma also impacts on clinical practice, as CHC nurses feel unable to communicate about children’s weight status for fear of being seen to participate in social stigmatization. This indicates that healthcare professionals may benefit from additional training on how to communicate with parents sensitively and non-judgmentally. It has been shown that parents tend to use alternative terms to describe their children as having overweight, such as “robust” or “big for their age” [14], and healthcare professionals might find it useful to use this terminology with parents. There is also considerable research on how to convey difficult messages in medical contexts; for example, Fallowfield and Jenkins [33] advise to show concern, confidence and care, have time for questions, prepare written information, value the child and respect the child and the parents. This reflects the foundations of good health communication that, according to the American Academy of Pediatrics [34] includes informativeness (quantity and quality of health information provided by the healthcare providers), interpersonal sensitivity (emotional behaviors mirroring the health care provider’s attention to the parents’ and child’s feelings and concerns) and partnership building (the healthcare providers invites the family to share their concerns, perspectives, and suggestions during the visit). To reduce the impact of weight stigma, CHC nurses can also increase parents’ awareness about the complexity underlying the development of overweight and obesity. In the parent support treatment program for preschool obesity that we have recently evaluated [13] the very first meeting is used to provide information on the role of genetics, individual susceptibility and the obesogenic environment in the development of obesity. This allows us to convey to parents that although they can now enact some changes to improve their child’s wellbeing, there is no room for guilt, blame or judgment where childhood obesity is concerned.

The second major finding from this study is that CHC nurses’ willingness to initiate conversations about children’s weights is hindered by limited cooperation with other healthcare providers. Partly, this is due to the fact that, while Sweden is one of the few countries in the world with universal routine assessment of weight status in children [35] no national guidelines for prevention and treatment of childhood obesity are established [8]. Nurses reported that pediatricians’ views on obesity treatment did not always match their own, such that children referred to the outpatient pediatric clinic were sometimes denied treatment. Similar finding emerged from a Swedish study conducted with CHC nurses in 2013, in the South of Sweden [19]. Isma and colleagues showed that the pediatric clinic sometimes rejected referrals concerning children with obesity, leaving the nurses to deal with the child’s weight management on their own. This was consistent with our findings. Furthermore, in our study, the nurses said it was more difficult to provide support to children who had overweight but not obesity, due to inconsistent guidelines within the health care system; for example, nurses were uncertain whether they could refer overweight children to treatment. This finding indicates that, at least in Sweden, treatment efforts are primarily focused on children with obesity care and should be extended to children with overweight.

To improve organizational capacity to address child overweight, nurses suggested that CHC centers should collaborate with preschools. This is highly relevant as 84% of Swedish children attend preschool [36]. Nurses also suggested that communication between professionals within the child health care setting should be strengthened; while the nurse is the key person, general practitioners/pediatricians, dietitians, physiotherapists and psychologists are a part of the treatment team [12]. This suggestion also accords with recent recommendations for a multidisciplinary approach to childhood obesity treatment [37]. Notably, none of the nurses in our sample said that children’s young ages were a barrier to obesity treatment, in contrast to Isma et al. [4], who found that most nurses didn’t “considered it meaningful to raise the issue with the parent if the child was 2.5 years or younger”. This indicates that an attitudinal shift with regard to early interventions may have taken place since 2012.

The participating nurses identified time constraints as another major obstacle to addressing children’s weights and offering families appropriate support. A limited number of CHC visits following the child’s first year, combined with limited time during the visits themselves (mainly due to administrative work and other competing priorities), limited nurses’ ability to discuss children’s weight development with parents. Time barriers are frequently cited as constraining healthcare professionals’ ability to address childhood overweight and obesity [14, 38, 39]. Several research reports highlight that childhood obesity is a long-term condition that requires continuous care [40] and that intensity of treatment (at least 26 contact hours) is one of the key factors to success [41]. Our research group has recently shown that an intensive program with follow-up booster sessions for preschoolers with obesity outperformed the same program without follow-up sessions in improving child weight status [13]. This suggests that routine appointments in the CHC setting should be structured strategically, so as to provide follow up on weight-related issues even if additional visits are not possible. For example, a routine visit that focuses on dental health advice can also include advice about eating habits, as both can benefit from for example reduced sugar consumption [42]. Lastly, the nurses said they would value continuing education within the area of childhood overweight and obesity as it would allow them to communicate confidently and advocate on behalf of their patients. Previous studies have found that parents often feel healthcare professionals downplay their concerns about their child weight or provide inadequate advice, underscoring the overall lack of training in childhood obesity among pediatric healthcare providers [21, 43]. In our study, nurses reported that their concerns about child overweight were often downplayed by pediatricians, a finding aligned with comprehensive surveys across Europe in primary healthcare, which show that most healthcare providers report they have insufficient knowledge on how to identify healthy child weight and provide guidance on nutrition, physical activity and psychological aspects related to eating and weight [39, 44, 45] It is important to emphasize, then, that lack of knowledge of childhood obesity is not unique to CHC nurses, but is an issue to address throughout the healthcare system. Indeed, experts have suggested that a specialization in childhood obesity should be established [8], in recognition of the high prevalence of obesity in Europe and elsewhere and the unique and still-unmet needs of this population [46].

### Strengths and limitations

A strength of the study is that many of the participating nurses worked in CHC centers located in areas with lower socioeconomic status and the highest prevalence of overweight and obesity in Stockholm County. One potential limitation is that, because participants were self-selected, nurses who were particularly interested in childhood obesity were more likely to take part, and it is therefore possible that the sample is not representative of all CHC nurses. We also recognize that some interview responses might have been influenced by social desirability bias, reflecting nurses’ understandings of what they were expected to think and say, rather than only their authentic experiences and perceptions. Finally, a particular strength of the study is that our findings share similarities with the international literature on communication between healthcare professionals and the parents of children with overweight/obesity. As this wider literature represents different countries and healthcare systems, this adds credibility to the results and highlights the global relevance of this issue.

### Future implications

While this study examined the perceptions of CHC nurses, it would also be valuable to conduct observation-based studies to analyze meetings between parents and CHC nurses. Parents’ perspectives on childhood overweight and obesity management from an early age should also be examined, and we plan to do so as a part of the More and Less Study Europe [25]. Triangulating CHC nurses’ and parents’ perspective would be an important step in developing recommendations for improving current organizational practices regarding weight management.

The findings indicate that socially stigmatizing attitudes and beliefs surrounding overweight and obesity inhibit early identification of excess weight among children. This suggests that public health initiatives should focus on reducing the stigmatization of overweight and obesity on the societal level; this could be done through a combination of a social marketing campaign aimed at reaching the wider public, and gatekeeper education (e.g. webinar sessions) aimed at schools and primary care. Another important finding is that the structure of the CHC needs to facilitate cooperation across clinics and between healthcare providers. In addition, CHC visits should be extended, to allow nurses more time to speak with families, and nurses should be offered continuing education and mentoring to support families with children with overweight or obesity, for example, through workshops built into nurses’ training and professional development curricula. Finally, CHC nurses’ confidence in communicating about childhood overweight and obesity will increase if they receive continuous guidance and communication skills training to navigate challenging conversations. Otherwise, change will be slow. As explained by a technical report from the American Academy of Pediatrics, “health care communication is learned primarily through trial and error”, which “may be attributable, in part, to a dearth of skilled mentors.” [34].

## Conclusions

In this study, we interviewed CHC nurses from 10 municipalities in Stockholm County, including four of the five most deprived areas, about their experiences of communicating with parents about child overweight and obesity. Across the sample, nurses identified young children’s overweight and obesity as sensitive topics to bring up in conversations with parents. The nurses identified several relational and organizational factors as barriers to discussing childhood obesity with parents. On the relational level, nurses emphasized that developing a trusting relationship with parents was essential to supporting families of children with overweight or obesity; at the same time, nurses often deferred or avoided conversations about children’s weights for fear that such conversations would compromise the trust they had built. Among the organizational obstacles, nurses identified lack of cooperation with other healthcare providers and with preschools as barriers to providing appropriate treatment. Organizational barriers also included time constraints and knowledge gaps regarding child weight management, which affected the nurses’ ability to support families and address weight stigma in conversation. The findings highlight that the social stigmatization of obesity plays a central role in silencing conversations between parents and healthcare providers and thus contributes to delays in treatment. This suggests that public health initiatives should focus on reducing the stigmatization of overweight and obesity on the societal level. Furthermore, the findings concerning organizational barriers suggest that healthcare systems should invest in offering continuing education about childhood obesity to clinicians and improve cooperation between clinics, gatekeepers and providers, in order to provide the families of young children with overweight or obesity the timely and optimal support they need.

## Supplementary information

**Additional file 1.** COREQ (COnsolidated criteria for REporting Qualitative research) Checklist.

## Data Availability

The transcripts/data of this qualitative study are not publicly available due to confidentiality agreements with the participants.

## Abbreviations

CHC: Child Health Care
STOP: Science and Technology in childhood Obesity Policy
